# Communication skills teaching and learning in Nepal; what are medical students’ perceptions and experiences? A qualitative study

**DOI:** 10.1186/s12909-020-02330-y

**Published:** 2020-10-29

**Authors:** Amanda Helen Douglas, Samita Pant Acharya, Lynne A. Allery

**Affiliations:** 1 Department of GP, Patan Academy of Health Sciences (PAHS), P.O.Box 26500, Lalitpur, Kathmandu Nepal; 2 Centre for Medical Education, Cardiff University, Heath Park, Cardiff, CF14 4YS UK

**Keywords:** Communication skills, Experiences, Medical education, Nepal, Perceptions, Students

## Abstract

**Background:**

Communication skills (CS) are vital for doctors. Indeed, as the most important element of consultations, are highly valued by patients. CS are core, teachable skills, however, have not been widely taught in South Asian medical schools, unlike their western counterparts.

Patan Academy of Health Sciences, is one of the first in Nepal to have CS central to its’ aims and curriculum. CS are taught from the first weeks of medical school and re-enforced during preclinical study (first 2 years). Our study seeks to explore students’ perceptions and experiences of CS teaching in this South Asian, Nepal context.

**Methods:**

This study is a qualitative evaluation of a CS course in Nepal, exploring the experiences and perceptions of participants. The study aims to also identify aspects that were helpful or not for student learning and areas for potential development.

A purposive sample of twenty: second, fourth and Intern year students was selected for interview. Data were collected through audio recorded semi-structured interviews following a piloted schedule. Interview transcripts were manually coded and thematically analysed. Codes were arranged into themes and subthemes.

**Results:**

The two main themes:

1. Positivity
2. Experiential learning.

Results demonstrate participants’ positive perceptions of CS teaching: believing it is important, effective, relevant and valuable for personal development. Participants identified experiential learning features as valuable for CS acquisition. Intern students recognised CS relevance and requested expanding teaching to clinical years,incorporating challenging communication scenarios.

**Discussion:**

This study shows that PAHS’ CS course is well perceived and valuable to learners. Experiential learning is powerful for CS development. Expansion of formal, structured CS teaching through all years in a spiral curriculum, should be considered. Violence towards doctors in South Asia is increasing. Students recognised CS teaching’s significance in addressing this.

**Conclusion:**

CS teaching,still in its’ infancy in South Asia, is a pressing issue for medical educators here. Our study provides evidence it is well perceived with positive impacts in this context, particularly when employing experiential learning. Medical schools in south Asia should be encouraged to incorporate and strengthen their CS teaching curriculum. .

**Supplementary Information:**

The online version contains supplementary material available at 10.1186/s12909-020-02330-y.

## Background

Our capacity to communicate is foundational to being human and underpins clinical practice, with doctors undertaking up to 300,000 consultations during their career [1].

Communication and interpersonal skills are core medical competencies for a doctor [2, 3]. Good doctor-patient communication (the focus of this study) positively impacts most areas of care including: patient understanding and recall, compliance with treatment, pain control, symptom resolution, psychological and functional wellbeing, together with patient and doctor satisfaction [4–8]. Indeed, communication is the “main ingredient in medical care” [6]. Conversely, the negative impacts of poor communication on patient understanding are well documented [9, 10]. Doctor-patient communication skills (CS) can be learnt and taught [11]. Most western medical schools’ curricula include CS [12, 13]. Patients identify doctors’ empathy in consultations as most valuable [14–16], however, this “core and teachable” element of CS [17] is often lacking [18, 19]. Clearly not all doctors develop the attitude and skills to communicate effectively [20]. This study seeks to explore students attitudes and perceptions to CS teaching in Nepal.

### South Asian context

The majority of CS research discussed was conducted in western countries, where CS teaching is established, thus it cannot be automatically extrapolated to the South Asian context of our study. Several social factors of South Asian culture influence its’ applicability here: hierarchical societies with doctors highly respected, social collectivism limiting individual autonomy and producing group decision making, patients’ use of traditional medicine, high patient loads and a large educational gap between doctors and patients [21–24]. All these cultural factors result here in a preponderance of paternalistic consultation style [23–25], with doctors dominating interactions, as opposed to the “partnership style”, taught in the west [23, 26]. Despite this, patients in non-western contexts value: “mutual understanding and trust” [23], receiving clear explanations and answers to questions [27, 28] with most preferring a partnership communication style [22, 24, 29]. To patients in Asia a partnership constitutes, not a dialogue of equals, but the doctor demonstrating a caring attitude [23].

Many South Asian medical schools overlook CS, focusing instead on a traditional biomedical model of medicine, with exceptions found in several general practice departments [22, 30]. Despite being identified as a core skill for Indian medical graduates for over 20 years [31], many Indian medical schools curricula have not incorporated formal CS teaching [32, 33]. In 2015 MCI introduced an attitude and communication module [34] to medical colleges across India [33]. However, persistence of a fragmented approach and lack of emphasis on CS training has resulted in sub-optimal CS and persistence of a paternalistic consultation style against patient preferences [23, 25].

### Nepal context

Nepal echoes the barriers to patient-centred care seen in South Asia generally: workforce shortages and high patient load [35, 36], hierarchical cast-based society [37] and a large educational difference between patients and doctors [38]. Little Nepal based research has been published on communication, however, two sentinel papers by Moore [22, 37] looked at aspects of patient centred care and communication in this context. Nepalese patients were found to be unconcerned with “power sharing” in consultations but highly valued full explanations, information, and doctors’ friendliness and respect [22]: features that doctors acknowledge are often lacking in Nepali consultations [37]. The Nepalese doctors participating in this study, believed seriously ill patients did not want information about their condition, contrary to patients’ expressed desire [37]. Moore concludes that CS must become a focus of medical teaching in Nepal.

CS teaching has been routine in western countries for many years, however the situation in Nepal remains embryonic. A 2006 report [39] found most Nepalese medical schools’ curricula, as in south Asia generally, did not contain CS, medical ethics or doctor-patient relationship. Subsequently the Nepal Medical Council has included CS within guidelines for undergraduate curriculum and medical graduate’s core competencies [40]. Several Nepali medical schools have incorporated CS teaching within: pharmacology [41], medical humanities [42] and basic sciences [43].

Patan Academy of Health Sciences (PAHS) in Nepal, founded in 2010, aims to train: “*technically competent, caring and socially responsible physicians … … .who*; *Believe in compassion, love, respect, fairness and excellence*. *Communicate well with patients, family and colleagues*”. CS are central to PAHS’ aims and curriculum. PAHS’ undergraduate course is divided into 2.5 years preclinical (basic science course) and 2 years clinical teaching, followed by a 1 year student internship. Formal structured CS teaching occurs throughout the pre-clinical years, within the Introduction to Clinical Medicine course (ICM). In the clinical years, these skills are reinforced and practiced during GP classes and clinical placements, building on good practice from CS teaching concepts and frameworks, however, there is no formal CS teaching during the clinical years. The pre-clinical CS curriculum includes: general communication skills, listening, information giving, breaking bad-news and dealing with angry patients. CS teaching is delivered by General Practice faculty members, as in most Asian countries [37], who have undertaken CS training*.* Teaching sessions comprise of lectures and roleplay as well as longitudinal and rural placements. Role-play sessions engage students in acting clinical scenarios, playing the parts of patient, doctor or relative. Student observers and faculty provide structured feedback. In longitudinal placements students are linked to: a patient with disability, chronic disease and palliative patients, visiting in their homes twice a month for 6 months. Students reflect on their experiences in a portfolio, exploring the impact of illness and disability, often discussing communication challenges observed [44]. For 2 months of the preclinical course and 5 months in the final clinical year, students are placed in rural locations to undertake community health projects and clinical clerkships in a district hospital. Preclinical students stay with local families during this placement. CS are assessed within both the preclinical and clinical years as a component of: OSCE (Objective Structured clinical examinations) comprising minimum 10% of questions, clinical evaluation exercises (Mini-CEX), direct observation of procedural skills (DOPS) and end of placement evaluations.

PAHS is a relatively new medical school and uniquely in Nepal, has CS central to both its aims and curriculum. PAHS students are all Nepali citizens, drawn from a diverse range of ethnic heritages and geographical areas of the country. Admission preference is given to students from disadvantaged backgrounds through a quota allocation: 10% from rural areas, with a weighting towards female or lower cast candidates, and 15% from lower socioeconomic groups. During our study persiod approximately 15% of students held full scholarships,(subsequently increased to 75%, due to a government program), 45% partial (half) scholarships and 40% paid full tuition fees (although some had private sponsorships). The ratio of male to female students is just under 2:1 in the study year groups. Nepali schools predominantly employ didactic teaching with minimal student participation [45]. In order to facilitate students’ transition to new teaching and learning styles, of interactive small groups, computer work and meeting patients, students completed a 6-month foundation block (reduced to 6 weeks, for subsequent student cohorts), comprising of skills, including basic communication awareness and knowledge.

This study is a qualitative evaluation of the PAHS CS curriculum, seeking to identify students’ perceptions and experiences of CS and CS teaching in this Nepali, South Asian context.

Students perceptions of learning are important and have been found to have a greater impact on learning than the teaching method used [46]. Perceptions affect how learners view materials, their learning approach [46–49] and outcomes [50]. Attitudes impact CS learning, implementation and use in practice [51–53], whilst positive perceptions are vital for learning and performance improvement [54]. Learners with negative perceptions of CS teaching may undervalue the skills and choose not to develop or use them in clinical practice [53]. Students attitudes and perceptions to CS teaching must be understood and if necessary, addressed to augment its’ impact [55].

Two Nepal based studies on voluntary CS courses [39, 56] show predominantly positive student attitudes, but with higher levels of negativity than seen in UK [57]. Both studies recommend strengthening CS teaching and expanding it into all Nepalese Medical schools. There are no published Nepal or South Asian based studies evaluating student attitudes or perceptions to core CS teaching, our study seeks to address this gap.

## Methods

This study employs qualitative methodology, underpinning interpretivist paradigm with subjectivist social construction and phenomenographic epistemology. This study was designed to explore a deep understanding of the subjective reality of student participants [58], their different experiences and perceptions. Semi-structured interviews were selected for data collection as they allow exploration of responses at depth and freedom to investigate ideas and themes [59], as interviewer and participant co-construct the interview [60].

### Participants

The study population was taken from PAHS medical students in Second year (pre-clinical course) and Fourth year (clinical course) and student interns (in their first-year post graduation).

Several year groups were sampled to capture a diversity of perspectives from those with varied levels of clinical exposure and proximity to formal CS teaching. Purposive sampling was employed. All students within study year groups (60 per year) received an email invitation to participate from their student representative, forwarded on from the researchers. The first email responders were interviewed until sufficient numbers were reached, determined by data saturation [61].

Twenty medical students from PAHS were interviewed, between February and April 2018: seven 2nd year students, seven 4th year students and six student interns .

### Ethical consideration

Ethical approval was gained from local ethics review board, prior to the study’s commencement. Participation was voluntary, informed written consent was taken and participants understood they were free to decline to participate or withdraw from the study prior to thematic analysis. Participants were assured their identity, views and opinions would be kept confidential. As a volunteer teacher at PAHS, the interviewer was known to many of the participants. However, her teaching of the study year groups was limited and she had not been involved in communications assessments or examinations. Extracts from interviews are reported using pseudonyms. Any identifying features were removed during transcription. Participants were made aware that we were interested in their views and there were no right or wrong answers, and all comments were welcomed. Participants understood that the interviews were part of a research project evaluating curriculum, may inform future medical education and would potentially be published.

### Reflexivity

As the research instrument [62, 63], the researcher’s perspective is integral to study findings [64]. Hence reflexivity was prioritised in our study, with the interviewer considering her feelings, thoughts, values and opinions in a field notes journal, and how these shape the data produced [65].

The interviews were conducted by the first author, a female, British expatriate doctor, resident in Nepal and a volunteer member of GP faculty supporting medical education and supervised as part of a Cardiff University postgraduate qualification. As a novice researcher, extensive preparation was undertaken prior to data collection and interview technique was practiced during pilot interviews.

### Data collection

Semi-structured interviews were employed. The schedule provided a guiding structure without rigidity, enabling flexibility and responsive questioning, allowing participants to raise unanticipated yet important issues [66]. The interview schedule (see Additional file 1) incorporated specifically written standard questions, employing “funneling”; with broad general, non-threatening early questions placing the participants at ease and aiding rapport building, with subsequent, more targeted questions [66]. All questions were open, non-leading and not assumptive [66], allowing scope to explore feelings or unanticipated issues [67], attitudes and opinions [68] and subjective experiences [69]. Basic counselling and GP communication skills of reflection and attending, helped make participants feel heard and more likely to share [70].

Twenty semi-structured face –to face interviews were undertaken at a time and place convenient to the interviewee. Each lasted between 20 and 45 min. Interviews were conducted in English, the medium of medical teaching in Nepal and the interviewer’s native language. The interview schedule was reviewed to ensure clarity and comprehensibility for non-native English language participants and then piloted in a non-study year group. Interviews were audio recorded and field notes were employed to record observations both during and after.

### Analysis

Interview recordings were manually transcribed by the interviewer soon after they occurred, to enhance credibility. All contextual and non-verbal interactions were retained and recorded in the transcript in an orthographic [66] method. Several participants used occasional Nepali words; a local translator ensured accuracy in English. A summary of each interview transcript was sent to participants, requesting comments on its’ accuracy, generating respondent validation [63]. Interview transcripts were manually coded by a single researcher, using semantic codes and thematically analysed (TA). Fragments of data allocated the same code were collected and compared, ensuring coding consistency. The researcher was immersed in the data, reading and rereading transcripts in order to understand the context and meaning, recoding segments in an iterative manner, as necessary, and ensuring coded fragments retained their original meaning. Different cases were compared during analysis, ensuring the interpretation and themes produced were representative of all participants’ voices. In particular negative cases were cited, to enhance credibility [71].

## Results and analysis

Twenty participant interviews were conducted, Table 1 details the demographics of  participants. The interviews generated 40 codes: five subthemes and two overarching themes see Table 2.

**Table 1:** Interview participants

Student year group	Male	Female	Total
**2nd**	5	2	7
**4th**	5	2	7
**Intern**	4	2	6
**Total**	14	6	20

**Table 2:** Analysis themes, subthemes and exemplar quotes

Themes	Subthemes	Examplar Quotes
1. Positivity	Importance of CS teaching and skills	*“it is very important to be able to communicate well to be a good doctor”,Khim, 4th year.*
	Relevance of CS teaching	*“what I have learned there … I started to use them in practical life … … instead I found it difficult but I thought it is more effective”, Nara, 4th year*
	Personal Development	*“I now started to feel confident … it have helped me a lot because I am changed”, Divesh, Intern*
2. Experiential Learning	Effective Role Playing	*“we have really learnt how to talk to a patient and about keeping us in their shoes … .we have learned a lot in the acting classes” Shova, 2nd year.*
	Relevant Rural Posting	*“we are sent in rural areas for posting so during this period we are exposed to strangers, so at that time … talking to them like slowly it builds up our communication skills” Arpan, 4th year.*

### Theme 1- positivity

This theme describes the value and worth attributed to CS teaching. Encompassing comments regarding: the importance, relevance and value for personal development, of the teaching and skills, positivity was identified in all the interviews. These were grouped under separate subthemes and discussed below.

#### Importance of CS teaching and skills

Asked about their feelings on CS, all participants mentioned its’ importance, particularly for becoming a “good doctor”:

***“****in the future it is very important to be able to communicate well to be a good doctor”* (Khim, 4th year).

One participant, explained CS teaching was valuable for a good doctor-patient relationship, which he believed was lacking in Nepal:

“*so, I think it really helps to establish that good doctor patient relationship which our country lacks … because doctors don’t communicate that well with their patients. So as a subject, being taught good communication skills at this level helps”,* (Sunil, 2nd year).

The majority of interviewees mentioned the importance of CS and CS teaching for reducing conflict and misunderstanding in medical settings**.** Several cited examples of medical conflict in Nepal, which they believed were caused by lack of CS:

“*in news there are so many now anger outburst … when the doctors get really physically abused and if we explored the things what happened then … there must be some kind of communication lack in those conditions* [hospital care] *and .. so, I think the skills that I have already acquired … will be very helpful in those situation*”, (Bibek, Intern).

All the study participants perceived CS teaching as generally important, with some identifying specific areas of patient care.

#### Relevance of CS teaching

Relevance subtheme encompasses descriptions of CS and teaching as having real-world value. The majority of participants identified CS as helpful in their practice with interns particularly appreciating CS’ relevance, possibly due to their greater clinical experience.

One student described questioning the relevance of CS teaching during pre-clinical years, but later appreciating its value:

[re CS teach]; *“we had many lectures on that and it looks simple but it was very necessary thing, I have realised now … we are applying it in our daily lives”,* (*Ram, 4th yea*r).

He continues later:

*“in basic sciences we may feel like “why we are learning this?”, “why? because … we are not even in clinical practice?” so maybe at that time students feel, why it is given emphasis? why it is more focused* [on]*. But now we realise* …”, (Ram, 4th year).

Several participants mentioned receiving positive feedback regarding their CS. One intern described a patient saying he was like a family member:

[re CS teaching]***:*** *“many patients praised me on my communication, they say, ‘so you are so trust worthy* [as] *if you have me as your family member’ and I was so glad”*, (Divesh, Intern).

CS were appreciated by patients but also lead to learners’ personal satisfaction and positivity that can be important for clinicians working in difficult circumstances.

#### Personal development

The value of CS for personal development was identified in many interviews. A number of participants described themselves as shy or introverted, struggling to express themselves and the value of CS teaching for developing those skills:

“*when you join PAHS, everyone sees us as doctors and we need to be able to communicate to patients … a lot of them* [students] *are extrovert they really can talk to anyone they want … but for some of us it is really difficult to express ourselves. so when I joined PAHS this* [CS teaching] *is something that I really appreciated..” (*Anup, 2nd year).

Nearly all interviewees described either an increase in their confidence communicating or improved CS:

[re own skills]: “*it has been changed, changed positively … before starting medical college I didn’t have much communication skills, I didn’t talk to the patients, anyone that good. but I have learned how to talk to them, how to be polite and how we should listen to them very carefully so they shouldn’t misunderstood it”,* (Yogya, 2nd year).

Several participants who described themselves as shy, mentioned how CS teaching helped increase their confidence communicating:

[re own skills] *“I am feeling confident with the patient conveying messages, asking them and even in* [with] *the colleagues … I now started to feel confident … it have helped me a lot because I am changed... from a shy person to an average communicator now so I feel great”,* (Divesh, Intern).

All intern participants identified the value of CS for difficult communication challenges faced during their clinical practice. Many experienced conflict and aggression:

“*one example of* [during] *the rural posting, there was a lot of problem regarding communication … many of the people I saw there tend to be aggressive towards health workers..”* (Krishna, Intern).

The majority of students described the relevance of CS teaching in their experience, recognising their personal development of skills and confidence and the necessity of such skills. Interns particularly articulated the value of CS teaching in their clinical practice.

The theme of positivity was seen across all groups of participants. Several subthemes, however, emerged more strongly from intern interviews, particularly: the importance of CS for reducing patient anger and violence, the value and relevance of CS teaching in their medical roles and the recognition of the positive impact of CS on patients and the resulting personal satisfaction. This is likely to result from interns’ greater clinical exposure and experience interacting with patients.

### Theme 2- experiential learning

Experiential learning is defined [72] as the construction of knowledge and meaning from real-life experiences. Learning ‘situated’ [72] in a context relevant to the learners’ future career. Whilst discussing areas of the CS curriculum they found effective, nearly all participants mentioned experiential learning features of role play and new learning contexts for example rural clinical posting.

#### Role-play

The majority of participants mentioned role-play as effective and helpful in CS learning. Role-play is an experiential learning technique in which case scenarios are acted out by learners [73].

The majority of participants expressed positive views on role play, preferring it to traditional didactic teaching. One described how it improved visualisation and retention of learning:

*“if it was just a class I would have already forgotten about it, since I was also a part of the role play I remember it more vividly … in a role play you can actually sit in the patients place and realise how you should actually communicate to them”,* (Shova, 2nd year).

Several participants commented that role-play allowed them to experience and prepare for real-life scenarios. Others, that it enabled them to appreciate patients’ feelings and develop empathy:

***“****in that role-play I really learned about if you were in the shoes of the patient what you would expect from the doctor … and what you would have liked”,* (Shova, 2nd year).

Several participants highlighted the value of breaking bad-news role-play learning.

“*there was a session of breaking bad news … we learned a little bit in class. we then practiced it, where we are actually given a check list for that and … people just went in the front tried to do that so it was seemed really helpful … we had to follow the palliative care* [patient] *just now …* [and] *I saw how much helpful it was. really it was really helpful”,* (Anup, 2nd year).

Many participants requested more opportunity for role-play practice of CS.

#### Rural posting

Many student participants identified the different context of the rural community posting as effective for learning CS, describing the opportunity to meet patients in their homes as valuable for skills and confidence development:

“*we are sent in rural areas for posting, during this period we are exposed to strangers.so at that time, talking to them, slowly it builds up our communication skills”* (Arpan, 4th year).

Another describes learning how to communicate and respect patients during their rural posting:

*“every year we were staying in the rural community posting for a week and our teacher … taught what we have to do... and how we have to communicate over there … giving respect … so I think this is very helpful”, (Rajesh,* 2nd year).

#### Ideas for change

Participants discussed changes they believed necessary to CS teaching or curriculum. Most requested more CS teaching:

*“I think there should be more classes on it* [CS]*, initially we had classes on many skills... as part of ICM we were being taught about, so as much as* [more] *classes on it would be likely helpful”* (Divesh, Intern)*.*

Requesting additional CS teaching, participants did not indicate how this could be achieved within a busy curriculum or what aspects could be omitted.

Many participants also identified the need for formal CS teaching during clinical years. One mentioned needing to refresh skills taught in earlier years:

*“I think initially there was adequate importance given to it* [CS] *so I was pretty satisfied... but in the clinical sciences there hasn’t been much talk on this communication skills. so maybe they thought that … they have already learnt it and we haven’t had any classes as such, especially focused on communication after we entered the 3*^*rd*^ *year … it would be beneficial if we keep refreshing it up”* (Khim, 4th year).

Several interns, however, felt that clinical years CS teaching was unnecessary as skills developed naturally through clinical interactions and placements:

[re CS Teaching] “*it is just enough, if we add some more things then it will be just waste of time*” (Rohan, Intern).

Clearly there is a need for CS teaching to be relevant to individual learning needs. Integrating CS teaching with clinical placements, particularly addressing communication challenges encountered would enhance its’ value.

The majority of interns and many 4th years believed that clinical years’ CS teaching was insufficient and requested formal or explicit CS teaching throughout clinical years. Many identified role-play learning as the most helpful for skills practice.

## Discussion

Learners’ attitudes towards CS teaching are powerful, influencing their perception of the importance of skills, their behaviour [74] and skills usage with patients [52, 53]. Our study participants had positive perceptions (proxy for attitudes) towards CS and CS teaching. They believed teaching to be helpful for increasing confidence and that the skills taught are important for medical practice, similar to other findings [53, 75].. Interns’ particular identification of teachings’ effectiveness and relevance reflect findings amongst new medical graduates [76]. .^]^ Medical students believe CS teaching is helpful for those needing CS improvement [77], seen in our participants, many of whom described themselves as shy or introverted. Data on the prevalence of shyness amongst Nepali medical students is lacking, however, several features of Nepaliand South Asian culture and school teaching may predispose students to feel shy in group interactions or meeting patients. Nepali schools, like many in South Asia, largely employ didactic teaching, with minimal student participation or interaction [45] and student silence in classrooms [78]. In a traditionally hierarchical, paternalistic and cast based culture [37], students from rural or disadvantaged backgrounds may feel reluctant or intimidated by the interactive elements of medical education. These students potentially benefit the most from CS teaching [77] and PAHS’ admissions weighting for such students, highlights the importance of reinforcing such teaching.

The real-life relevance of CS, identified particularly by interns is usually only recognised after commencing clinical placement [79], possibly due to a positive attitudinal shift towards CS teaching [55]. Consequently, CS teaching should be situated in and integrated with clinical experience [77, 80], in agreement with our participants’ request for increased clinical years CS teaching. CS teaching often has low priority in medical schools [81] and there is debate regarding its’ timing. Pre-clinical CS teaching provides a safe environment for skills practice prior to patient exposure [82], however, skills deteriorate without reinforcement [81]. One review concluded CS teaching is more effective during clinical years [11]. Most research supports starting CS teaching early, to raise awareness and understanding of the key tenants of CS for clinical practice, and reinforcing it regularly throughout clinical years, in a spiral model, [11, 83] which leads to better skills attainment [83]. In order to enhance students’ CS acquisition during clinical years, clinical and educational supervisors should be encouraged to provide feedback and support on CS when observing trainees on placement, providing relevant, situated CS feedback and learning. Perhaps PAHS should review the weight of CS teaching in the medical curriculum, particularly clinical years. Considering increasing formal CS teaching for clinical students, providing opportunities for them to refresh skills, building upon earlier CS learning by integrating it with their clinical experience. Ensuring the central place of CS within the curriculum, mirroring its’ explicit integration within learning outcomes, competencies and end of placement reviews.

Active learning is superior to passive [84] and adults learn best through interactive, relevant teaching [82]. Experiential learning, the construction of knowledge from real-life experiences [72] is active learning. Our participants particularly identified two experiential learning features as helpful: role-play and rural placements, aligning with students known preferences for experiential teaching methods [73]. Role play enables specific, CS practice [85] and is the most widely used method for CS teaching [86]. It is effective for CS teaching with learners extemporising interpersonal and professional behavior, [ [84] and improving skills [87]. By providing a safe environment for skills practice [73, 84], role-play can ease transitions into clinical settings [88] and can be useful later in training to address more targeted needs, based on real scenarios. Didactic teaching is useful for increasing knowledge about CS [89], in particular awareness of professional standards and good practice, but is ineffective compared to experiential methods [11], particularly roleplay [90]. Students benefit from repeated opportunities for skills practice [82] with feedback, as requested by our learners.

Medical educators should consider reviewing teaching methods employed for CS, particularly the proportion of didactic, lecture teaching to experiential learning. Ensuring that teaching modalities are constructively aligned with intended learning outcomes [91]. Expansion of roleplay learning should be considered, particularly within the clinical years, incorporating challenging scenarios identified by interns, such as angry patients.

The rural posting, another form of experiential learning, was identified s as particularly effective for CS. Students value opportunities to interview real patients, preferring learning CS through experiential means in clinical settings [73]. Essential to experiential learning is students’ reflection on their experience [92]. This facilitates the transformation of experience into learning, enabling its’ integration and contextualisation, and facilitating skills improvement [93–95]. Rural medical practice in Nepal faces many challenges including lack of infrastructure, equipment and personnel [96] which may hinder effective communication. Learners’ should be encouraged to reflect upon their rural experiences through discussion with clinical and educational supervisors within placements as well as in their written portfolios, enabling their own critical evaluation of experiences and potentially inspiring them to better communicators [44].

This study was conducted in Nepal, however, the findings are of interest to medical educators across South Asia, with similar cultural contexts and embryonic CS teaching.

The 2015 MCI introduction of competency-based CS teaching, within the ATCOM module, in Indian medical schools is an encouraging development, after many studies highlighted this as an urgent need [31]. ATCOM incorporates many of the features discussed above, with longitudinal integrated CS teaching from first to final years and a combination of both experiential learning and explicit teaching [97]. The module has elicited mixed responses, particularly regarding its’ lack of formative skills assessment or evaluation [32]. One report explains how, without full inclusion of CS teaching within both medical curriculum and examinations, the module will not adequately address India’s CS issues [32]. Indian medical students’ attitudes are predominantly focused on knowledge acquisition and passing examinations, rather than skills acquisition [97]. Researchers argue that these need to change in order to facilitate CS development and incorporation into practice [97]. There is no published evaluation of students’ attitudes and perceptions of the ATCOM module, however, our study offers encouragement for this South Asian context. Our students’ and particularly interns recognised the relevance and effectiveness of CS teaching, indicating their likelihood of incorporating these skills into their medical practice [52, 53].

Another criticism of India’s ATCOM is its’ implementation by existing medical school faculty [32]. Teaching of CS requires different approaches than other clinical subjects [98]. There is concern that the written competency-based CS curriculum maybe different from that implemented, as observed recently in Indonesia [99] where faculty struggled to teach patient centred topics like CS [24]. Clearly curriculum change alone is not sufficient, medical educators need to ensure that faculty are sensitized, prepared for, and supported in, developing new teaching skills, a process not yet started in the majority of Indian medical institutions [32, 97, 100].

Violence towards doctors by patients and relatives is an increasing problem, not only in Nepal but wider South Asia [25, 96, 101], causing fear and stress particularly for rural doctors [102]. Although the reasons for this are multifactorial, poor doctor patient communication is a contributor [25, 102], identified by our students and needs to be adequately addressed through CS teaching.

## Conclusion

Although this study was conducted in Nepal the findings are potentially relevant to medical educators in other south Asian contexts where CS teaching is embryonic.

Learners’ perceptions of CS and CS teaching matters, impacting the value placed on communication and future care offered to patients [51]. Our participants positive perceptions of CS teaching are encouraging, however, it is important that thetiming of such teaching is reviewed, as skills acquired early in medical school, deteriorate without reinforcement. Medical educators need to refresh and build upon early CS teaching during clinical years, to prevent such a decline.

Experiential learning elements of curriculum were particularly important and effective, for CS learning. Increasing the proportion of experiential learning opportunities within curriculum should be prioritised. Educators should explore ways to facilitate students’ in -depth reflection on experiential learning, enabling critical reflection and evaluation of their own values to promote worthwhile change.

CS teaching should be encouraged and expanded throughout medical schools in Nepal and South Asia. This study shows it is well perceived by learners in this context and of particular importance given minimal CS learning in didactic school teaching and growing violence towards doctors.

### Study limitations and biases

Study interviews were conducted in English, participants’ second language and whilst English is used throughout training, participants may feel more comfortable speaking their native language when discussing feelings and opinions. Data analysis was performed by a single researcher but results were discussed by the research team and within tutor supervision. The study sample size was small, however, data saturation, the point at which adding more data does not create new themes, was taken as indicating sample sufficiency [61] for our study purposes. Areas of potential future research would be to explore the perceptions of clinical placement supervisors and trainers and their readiness to incorporate CS more explicitly within their feedback to trainees.

## Supplementary Information

**Additional file 1.** Semi- Structured Interview Schedule – 4th Year Students.

## Data Availability

The datasets used and/or analysed during the study are available from the corresponding author on request.

## Abbreviations

CS: Communication skills
DOPS: Direct observation of procedural skills
Mini- CEX: Clinical evaluation exercises
OSCE: Objective Structured clinical examinations
PAHS: Patan Academy of Health Sciences
TA: Thematic Analysis
