# Supplementary material for: Communication skills teaching and learning in Nepal; what are medical students’ perceptions and experiences? A qualitative study
Source: BMC Med Educ. 2020 Oct 29;20:391. doi: 10.1186/s12909-020-02330-y (PMC7596984; doi:10.1186/s12909-020-02330-y)
Supplement: Supplementary file 1 — Additional file 1. Semi- Structured Interview Schedule – 4th Year Students. [file 12909_2020_2330_MOESM1_ESM.docx]

**Additional file 1**

**Semi- Structured Interview Schedule – 4th Year Students**

Welcome and Introduction

Explanation of the study – Read through Participant Info leaflet together.

Written Consent sought. Start audio recording

**Introduction Qs** (Main questions by bullet point, clarification prompts underneath)

- **Q1;** What does the term Communication Skills mean to you?

How would you define it? How do you feel about it?

Is it an area that you are interested in?

**Communication skills Training – experience, feelings, ideas, attitudes and perceptions**

- **Q2;** Communication skills as a subject in medical school is new in Nepal. Tell me what you think about it.

importance? Why and in what ways? Relevance?

- **Q3;** Some people have said that communication skills teaching is a waste of time. What do you think about that?
- **Q4;** Others say that it is not needed as it is all common sense and you pick it up anyway. What is your opinion of that?
- Today I want to ask you about your experience of communications skills learning here at medical school.
- **Q5;** Please think of a specific, particularly good, effective communications learning incident you experienced. I want you to describe this incident to me in detail, including your role and what you felt or thought during it. Then the outcome of the incident. (Pause to allow participant to think)

Ok please can you tell me about the incident.

- **Q6;** Now I would like you to think of a specific, particularly ineffective communications learning incident and describe it to me in the same way. (Pause to allow thinking).

Ok, please can you tell me about the incident.

Can you be more specific,

What did you do? What were you thinking? How did you feel?

What was the outcome?

Do you identify any learning from this?

Does this incident resonate with any of your formal communications skills teaching? In what way?

What made it particularly in/effective

**Communication skills preparation/future use**

- **Q7**; How do you view your own communications skills?

Have they changed over time? Confidence communicating?

Has that changed over the course? In what ways?

- - - **Q8;** Are there any areas of communication skills that you feel have not been taught well?

Areas where you feel unprepared for your future role?

What was not effective about this?

- **Q9;** Are there any particular communication skill areas that you think have been well taught?

What made that teaching effective?

- **Q10;** What do you think about the relevance of the communication skills course to your (future) medical career?

In what ways was/is it useful in communicating on with pts and relatives/colleagues?

Do you feel prepared/equipped for communicating with others in work?

Do you think it will help you professionally? In what ways?

**Closing Question;**

- **Q11.** I think that is all I wanted to ask you about this. Do you have anything else you would like to say about the subject before we conclude our interview?
